# A novel reporter for real-time, quantitative imaging of AKT-directed K63-poly-ubiquitination in living cells

**DOI:** 10.18632/oncotarget.24323

**Published:** 2018-01-25

**Authors:** Shyam Nyati, Nauman Chaudhry, Areeb Chatur, Brandon S. Gregg, Lauren Kimmel, Dheeraj Khare, Venkatesha Basrur, Dipankar Ray, Alnawaz Rehemtulla

**Affiliations:** ^1^ Department of Radiation Oncology, University of Michigan, Ann Arbor, MI-48109, USA; ^2^ Life Sciences Institute, University of Michigan, Ann Arbor, MI-48109, USA; ^3^ UMCCC Proteomics Shared Resource, University of Michigan, Ann Arbor, MI-48109, USA; ^4^ Department of Pathology, University of Michigan, Ann Arbor, MI-48109, USA

**Keywords:** AKT, K63-ubiquitination, reporter, luciferase imaging, real-time

## Abstract

Post-translational K63-linked poly-ubiquitination of AKT is required for its membrane recruitment and phosphorylation dependent activation in response to growth-factor stimulation. Current assays for target specific poly-ubiquitination involve cumbersome enzymatic preparations and semi-quantitative readouts. We have engineered a reporter that can quantitatively and in a target specific manner report on AKT-directed K63-polyubiquitination (K63UbR) in live cells. The reporter constitutes the AKT-derived poly-ubiquitination substrate peptide, a K63 poly-ubiquitin binding domain (UBD) as well as the split luciferase protein complementation domains. In cells, wherein signaling events upstream of AKT are activated (e.g. either EGFR or IGFR), poly-ubiquitination of the reporter leads to a stearic constraint that prevents luciferase complementation. However, upon inhibition of growth factor receptor signaling, loss of AKT poly-ubiquitination results in a decrease in interaction between the target peptide and the UBD, allowing for reconstitution of the split luciferase domains and therefore increased bioluminescence in a quantitative and dynamic manner. The K63UbR was confirmed to be suitable for high throughput screen (HTS), thus providing an excellent tool for small molecule or siRNA based HTS to discover new inhibitors or identify novel regulators of this key signaling node. Furthermore, the K63UbR platform could be adapted for non-invasive monitoring of additional target specific K63-polyubiquitination events in live cells.

## INTRODUCTION

Eukaryotic cells employ a wide repertoire of ubiquitin (Ub) post-translational modifications to regulate biological processes. Conjugation of ubiquitin to protein substrates within a signaling cascade requires ubiquitin activating (E1), conjugating (E2) and ligating enzymes (E3). K48 mediated poly-ubiquitination leads to proteasome mediated substrate degradation [1], whereas K63 mediated poly-ubiquitination mediates many non-degradative functions, including enhanced protein-protein interaction, sub-cellular localization and kinase activation [2–5].

Dysregulation of E3-ubiquitin ligase activity is associated with many pathological processes including oncogenesis and chemo-resistance and provide novel targets for the treatment of human cancers [6–9]. Although our understanding of K63-linked poly-ubiquitination and de-ubiquitination in cell signaling is emerging [10–14], technologies to specifically monitor this regulatory event in normal physiology and in disease lagged behind, thus impeding the development of small molecules that inhibit this important class of regulatory enzymes.

The Ser/Thr kinase AKT (PKB) is a key hub in the cellular signaling response to growth factor and cytokine receptor activation and mediates biological functions including cell growth, cell survival and therapeutic resistance. AKT hyperactivation and mutation are common in cancer [15–17] and anti-cancer therapies have been directed against its kinase activity [18–20]. A role of K63-linkage specific poly-ubiquitination of AKT for its recruitment to the plasma membrane and subsequent kinase activation is well established [5, 11, 12, 21–25]. Inactivation of AKT requires its deubiquitination by deubiquitinases (DUBs) such as cylindromatosis (CYLD) [26–28]. Inhibition of AKT poly-ubiquitination therefore provides a unique opportunity for therapeutic intervention. Several E3-ubiquitin ligases have been investigated as therapeutic targets [6, 7, 29] and E3-ubiquitin ligase specific inhibitors have been developed [30], demonstrating the feasibility of therapeutic targeting AKT by inhibiting its poly-ubiquitination. Here we describe the engineering of K63UbR, a reporter for AKT-specific K63 poly-ubiquitin E3 ligase activity. Based on the split luciferase complementation technology, wherein AKT-directed K63-polyubiquitination activity leads to polyubiquitination of K63UbR which results in the interaction of the target peptide with the adjacent ubiquitin binding domain (UBD). This interaction restricts the complementation of luciferase, however, inhibition of target specific ubiquitination activity relieves this stearic constraint, allowing for luciferase complementation and enhanced bioluminescence activity. The reporter provides a sensitive, quantitative and dynamic non-invasive imaging of AKT poly-ubiquitination in live cells, and can be used as a research tool to delineate novel mechanisms that regulate the AKT signaling hub and also as a platform for high-throughput screening.

## RESULTS

### Domain structure and mechanism of action of K63UbR

**Figure 1:**
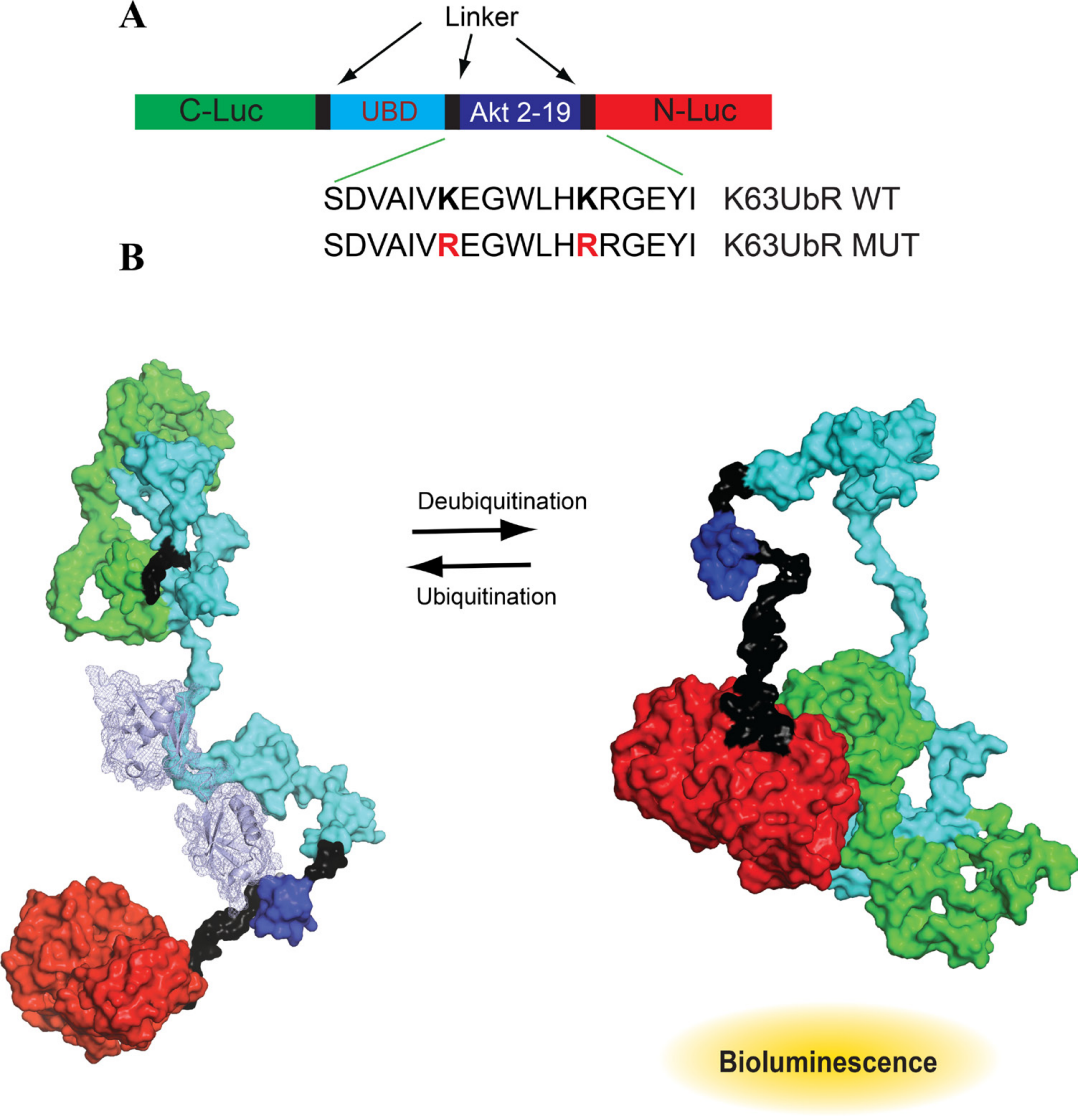
Design and mechanism of K63-linkage specific reporter (K63UbR) (**A**) Domain structure of the K63UbR construct: Two versions of the K63UbR construct were developed; the K63UbR-WT construct contains the wild-type AKT sequence, aa 2-19, and the K63UbR-MUT construct in which both the lysines were substituted to arginine. (**B**) The space filled model shows 3D domain structure of the K63UbR. The dark blue domain is the AKT substrate peptide (residues 2-19) encompassing K8 and K14. The mechanism of action of the K63UbR reporter involves E3-ubiquitin ligase dependent ubiquitination of the AKT target peptide which results in its interaction with the ubiquitin binding domain (UBD). The crystal structure of K63-linked di/polyubiquitin chains are known (PDB 2JF5 and 3HM3; [33, 67]). We docked the K63-linked polyubiquitin chain (cyan light) on the AKT substrate peptide manually. To avoid complexity a chain containing only two ubiquitins are docked on the AKT peptide. In this form, the reporter has minimal bioluminescence activity. In the absence of the E3-ubiquitin ligase activity, the target peptide is not ubiquitinated, it no longer interacts with the UBD domain allowing the N-Luc and C-Luc domains to reassociate, restoring bioluminescence activity.

K63-linked poly-ubiquitination of AKT is essential and a prerequisite for its membrane recruitment and activation [5, 11, 12]. Residues K8 and K14 within the Pleckstrin Homology (PH) domain of AKT undergo K63-linked ubiquitination by NEDD4 and TRAF6 in response to insulin like growth factor-1 (IGF-1) [10, 31] and by SCF^SKP2^ complex in response to epidermal growth factor (EGF)[32]. Residues 2-19 of AKT encompassing K8 and K14 were used as the target substrate sequence for the construction of K63UbR. Several K63-linked tandem ubiquitin interaction motifs (tUIMs) with varying degrees of selectivity have been identified [33–37]. We selected tUIM (amino acids 2-200) from Trabid (also known as ZRANB1) containing three tandem Npl4 related zinc finger (NZF) motifs as the ubiquitin binding domain (UBD), to achieve high specificity and selectivity for K63-linked poly-ubiquitin chains [37–40]. These sequences were flanked by the N-terminus of luciferase (N-Luc, 4-354) at the carboxyl-terminus of the reporter while the C-terminus of luciferase (C-Luc, 358-544) at the amino-terminus of the reporter. We named this engineered reporter as the K63UbR (Figure 1A).

The proposed mechanistic basis of the K63UbR reporter is as follows: upon activation of upstream signaling (growth factor treatment, e.g. EGF or IGF-1), the AKT specific E3-ubiquitin ligases (SCF^SKP2^, TRAF6, NEDD4-1) are engaged, leading to K63-linked poly-ubiquitination of the AKT substrate peptide which results in its interaction with the ubiquitin binding domain (UBD). This intramolecular interaction restricts the reconstitution of N-Luc with C-Luc and results in diminished bioluminescence activity (Figure 1B**,** left). Inhibition of AKT-specific E3 ligase activity relieves the interaction between the UBD and the target peptide, allowing the N-Luc and C-Luc domains to re-constitute, thus restoring bioluminescence activity (Figure 1B**,** right). To demonstrate the specificity of the reporter, we mutated the target Lys (K) in the AKT substrate peptide to create a mutant reporter (Figure 1B, K63UbR-MUT).

### K63UbR measures EGFR mediated K63-linkage specific poly-ubiquitination of AKT

**Figure 2:**
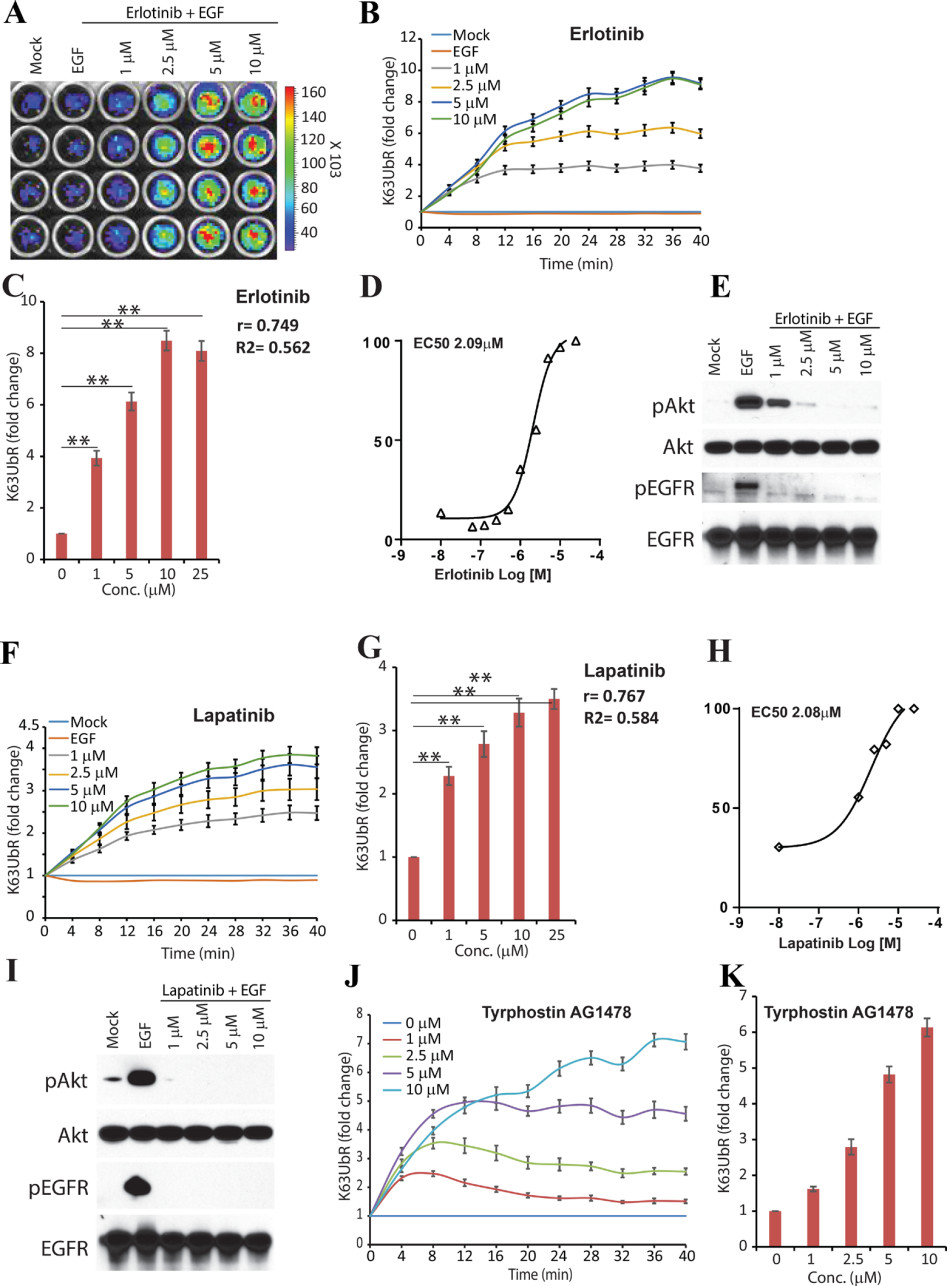
K63UbR reporter response to EGF and inhibitors (**A**) Reporter expressing MDA-231-1833 cells were treated with EGF or increasing concentration of EGFR inhibitor Erlotinib. A representative image of an area of 96 -well plate is shown. (**B**) Reporter expressing MDA-231-1833 cells were treated with EGF and Erlotinib and sequential bioluminescence was acquired four minutes after addition of growth factors which leads to a dose and time dependent increase in K63UbR activity. (**C**) Reporter fold induction with Erlotinib concentration is plotted to illustrate dose-dependent increase in the reporter activity. Pearson correlation coefficient (r), goodness of fit (R2) and statistical significance (*p*, ^*^<0.05, ^**^<0.001) is calculated and shown on the plot. (**D**) MDA-231-1833 K63UbR WT cells were treated with EGFR inhibitor Erlotinib to estimate the EC50 values in live cells under physiological conditions. The data plotted are from at least three independent experiments. (**E**) Cell lysates extracted in parallel experiments show a decrease in EGFR and AKT activation following treatment with Erlotinib. (**F**) A dual tyrosine kinase inhibitor Lapatinib which inhibits EGFR and Her2 activities, also lead to a dose and time dependent increase in K63UbR activity. (**G–H**) The dose dependency and EC50 values of K63UbR to Lapatinib was estimated from at least three different experiments. (**I**) Reporter expressing MDA-231-1833 cells were treated with EGF or increasing concentration of Lapatinib show a decrease in EGFR and AKT activation. (**J–K**), MDA-231-1833 K63UbR WT cells were treated with increasing concentrations of receptor tyrosine kinase inhibitor Tyrphostin AG1478 and bioluminescence was acquired for 40 minutes. A dose dependent increase in the reporter activity was observed.

Inhibition of growth factor signaling by small molecule kinase inhibitors has been shown to reduce K63-linkage specific poly-ubiquitination and activation of AKT [28, 31, 32]. Therefore, the sensitivity of the reporter was evaluated by plating the MDA-231-1833 cells stably expressing the K63UbR-WT reporter in 96-well plates and treated with increasing concentrations of the EGFR inhibitor, Erlotinib, (Figure 2A, 2B) or EGFR/Her2 dual inhibitor Lapatinib (Figure 2F**),** in the presence of EGF stimulation. Reporter activity was calculated as fold change over vehicle (control, DMSO) treatment. Since Lys63-linkage specific poly-ubiquitination and activation (phosphorylation) of AKT has been shown to occur within minutes in response to growth factor treatment [31, 32], we monitored the reporter response serially from 4 minutes to 40 minutes. The AKT inhibition using the above inhibitors led to a robust increase in the bioluminescence signal at the earliest time-point measured (4 minutes, Figure 2A, 2B and 2F) demonstrating that intra-molecular luciferase complementation within the K63UbR is immediate and results in a sustained response. Furthermore, to biochemically validate that inhibition of AKT ubiquitination leads to reduced AKT activation, cell extracts prepared in parallel experiments were probed using phospho-AKT specific antisera (Figure 2E, 2I). A decrease in phosphorylated AKT further validate the sensitivity of the K63UbR reporter. Time (Figure 2B, 2F) and dose dependent (Figure 2C, 2G) increase in the K63UbR activity was observed demonstrating the sensitive and quantitative nature of the reporter. The EC50 values estimated in live cells were 2.09 µM for Erlotinib, and 2.08 µM for Lapatinib, (Figure 2D, 2H). A significant (*p*-value), goodness of fit (R2) and correlation coefficient (r) between reporter fold activation, time and inhibitor concentrations further demonstrate that the reporter is very robust, sensitive and dynamic. In addition, the reporter expressing cells were also treated with an increasing concentration of receptor tyrosine kinase inhibitor Tyrphostin AG1478 which also demonstrated a dose dependent activation of K63UbR (Figure 2J, 2K) further confirming the sensitivity of the reporter.

### K63UbR measures IGF-1R mediated K63-linkage specific poly-ubiquitination of AKT

**Figure 3:**
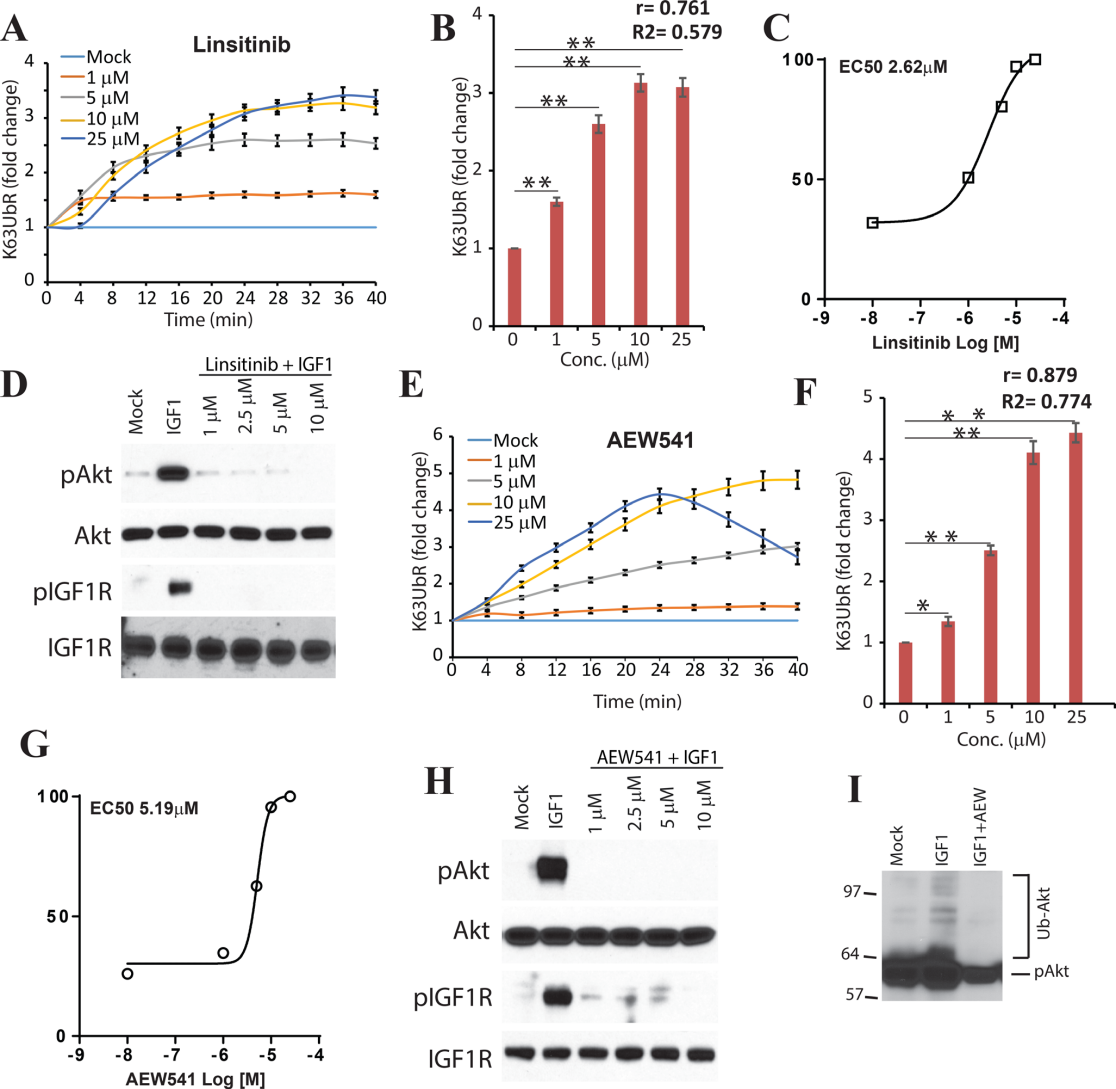
K63UbR reporter response to IGF1 and IGF1R inhibitors (**A**) K63UbR expressing MDA-231-1833 cells were treated with IGF1 or IGF1R inhibitor Linsitinib and sequential bioluminescence was acquired for up to 40 minutes after addition the luciferin substrate. The change in bioluminescence activity over mock treatment (vehicle) levels were calculated and plotted as fold induction (from quadruplicates). Data is representative of at least three different experiments. The K63UbR exhibits a dose and time dependent increase in response to IGF1R inhibitor Linsitinib. (**B**) K63UbR reporter fold induction with various concentration of Linsitinib is plotted to illustrate a dose-dependent increase in the reporter activity. Pearson correlation coefficient (r), goodness of fit (R2) and statistical significance (*p*, ^*^<0.05, ^**^<0.001) is calculated and shown on the plot. (**C**) MDA-231-1833 K63UbR WT cells were treated with IGF1R inhibitor Linsitinib to estimate the EC50 values under physiological conditions in live cells. The data plotted are from at least three independent experiments. (**D**) Cell extract prepared from K63UbR expressing MDA-231-1833 treated with IGF1 and increasing concentration of Linsitinib show a decrease in IGF1R and AKT activation following treatment. (**E**) Treatment of K63UbR expressing reporter cell line with increasing concentration of AEW541, another IGF1R inhibitor also lead to a dose and time dependent activation of the K63UbR. (**F**) K63UbR reporter response to various concentration of AEW541 is plotted to demonstrate dose-dependency of the reporter activity. (**G**) MDA-231-1833 K63UbR WT cells were treated with IGF1R inhibitor AEW541 and the reporter response was measured and combined from at least three different experiments to estimate the EC50 values. (**H**) Lysates made in parallel experiments show inhibition of IGF1R and AKT activities with AEW541. (**I**) MDA-231-1833 K63UbR WT cells were starved and treated with IGF-1 (50 ng/mL) or IGF-1 and AEW541 (10 µM) for 30 min. Cell extracts were prepared in urea lysis buffer and run on a SDS-PAGE gel and probed using phospho-AKT antibody.

Since IGF-1 mediated activation of AKT is known to occur through K63-linked ubiquitination [10, 31], the sensitivity of the reporter was also evaluated using IGF-1R inhibitors Linsitinib and AEW541 upon IGF-1 stimulation (Figure 3A, 3E). Treatment with the above inhibitors led to a robust increase in the bioluminescence signal as early as 4 minutes (Figure 3A, 3E) further confirming the sensitivity and dynamic nature of the reporter. Similarly, a time (Figure 3A, 3E) and dose dependent (Figure 3B, 3F) increase in the K63UbR activity was observed demonstrating the sensitive and quantitative nature of the reporter. The EC50 values were estimated to be 5.19 µM, and 2.62 µM for AEW541 and Linsitinib respectively (Figure 3C, 3G). Next, we investigated if the observed changes in reporter activity correlated with AKT activation using biochemical techniques (Figure 3D, 3H). A decrease in AKT phosphorylation was observed which further validated the sensitivity of the K63UbR reporter. To demonstrate that loss of AKT poly-ubiquitination was occurring when reporter activity was maximal, MDA-231-1833 K63UbR WT cells were starved and treated with IGF-1 (control) or IGF-1 in the presence of an IGF-1R inhibitor, AEW541. Cellular extracts were prepared in urea lysis buffer, resolved on SDS-PAGE gels and probed using a phospho-AKT antibody which showed that the AKT was poly-ubiquitinated in IGF-1-dependent manner which was abolished upon IGF-1R inhibition by AEW541 (Figure 3I) further confirming the sensitivity and specificity of the reporter.

### K63UbR specifically measures allosteric AKT inhibition

**Figure 4:**
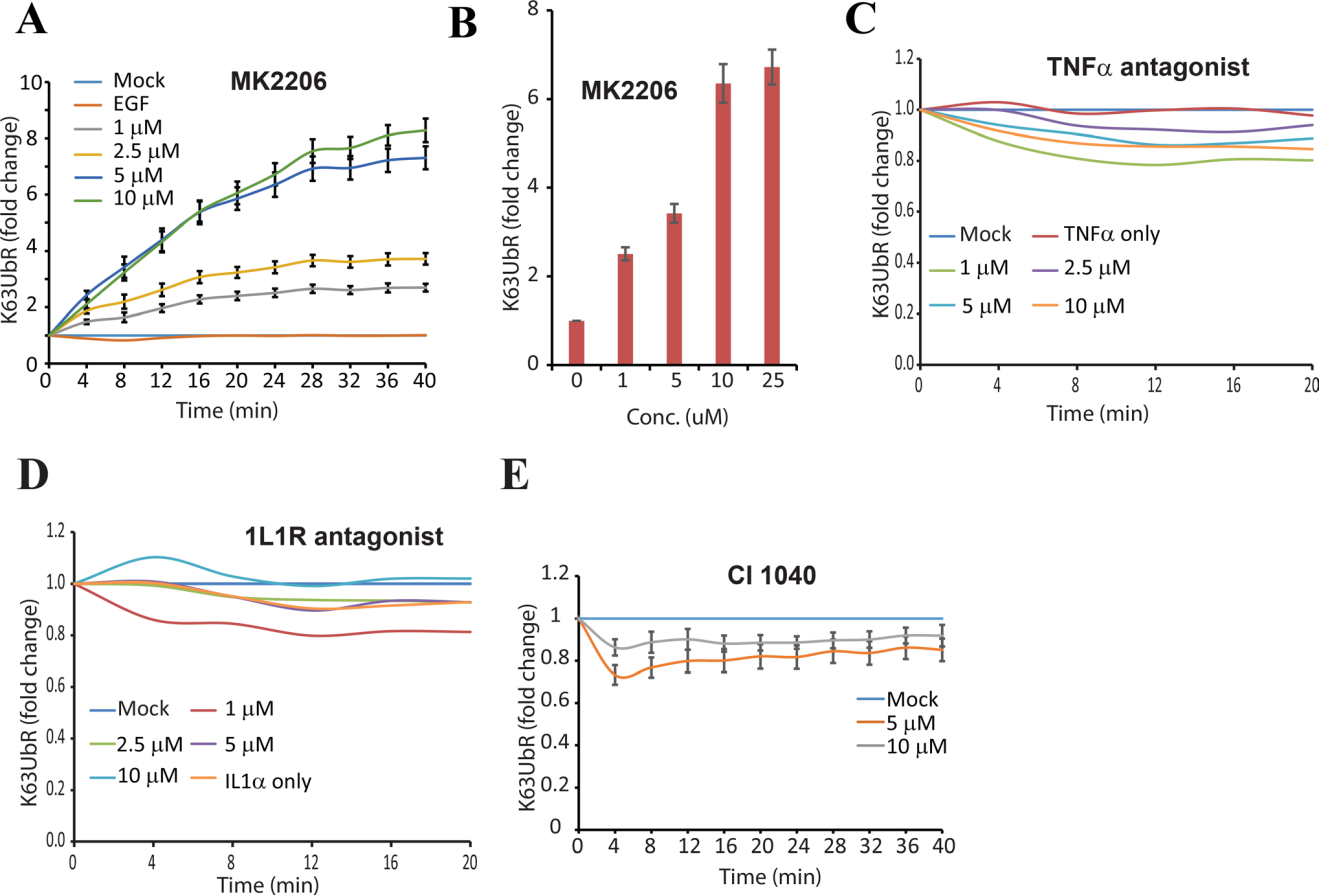
Validation of the specificity of the K63UbR sensor (**A–B**) To test the specificity of the K63UbR in monitoring AKT activation, MDA-231-1833-K63UbR cells were treated with an allosteric AKT inhibitor MK2206. Treatment with MK2206 resulted in a dose and time dependence increase in K63UbR activity. (**C**) K63UbR WT expressing MDA-231-188 cells were treated with vehicle (mock), TNF-α or increasing concentrations of TNF-α antagonist (WP9QY) in the presence of TNF-α and bioluminescence was acquired serially for 20 minutes. Reporter did not exhibit any change in the activity either in response to TNF-α alone or with its antagonist. (**D**) K63UbR expressing cells were treated with IL-1α or increasing concentrations of IL-1α antagonist (CAS No. 566914-00-9). The reporter did not show any significant change in the activity upon these treatments. (**E**) Reporter expressing cells were treated with MEK inhibitor (CI-1040) and bioluminescence was acquired serially from 2–40 minutes. As expected cells exhibited a decrease in reporter activity in response to CI-1040.

Allosteric inhibition of AKT (by MK2206) has been shown to block NEDD4 dependent K63-linked AKT poly-ubiquitination [31] as well as degradation of the AKT E3-ubiquitin ligase SKP2 [41]. Therefore, the sensitivity of K63UbR was evaluated in the presence of MK2206. MDA-231-1833 K63UbR cells treated with increasing concentrations of MK2206 demonstrated a dose and time dependent increase in K63UbR activity (Figure 4A, 4B) further confirming the specificity of the reporter.

### K63UbR does not respond to TNFα or IL1-R inhibition

Tumor Necrosis Factor-alpha (TNF-α) and Interleukin 1-receptor (IL1-R) pathways are known to engage TRAF6 (capable of polyubiquitinating AKT) [42], although these signaling events do not result in AKT activation (and poly-ubiquitination) [42]. Therefore, the specificity of the K63UbR reporter was further confirmed using TNF-α and IL1-R antagonists. As expected there was no significant increase in the K63UbR bioluminescence with TNF-α or IL1-R antagonists (Figure 4C, 4D). In comparison to K63UbR response to EGFR, IGF-1R inhibitors or AKT allosteric inhibitor (Figure 2B, 2F, 2J, 3A, 3E, and 4A), the reporter response to TNF-α or IL1-R antagonists (Figure 4C, 4D) is statistically insignificant at each dose or time evaluated.

Because K63-linkage specific poly-ubiquitination of AKT is prerequisite for its activation and MEK inhibitors have been shown to activate AKT in various breast cancer cell lines [43], we evaluated the sensitivity of K63UbR using a MEK inhibitor (CI-1040). As expected, the inhibition of MEK by CI-1040 led to a decrease in K63UbR activity (Figure 4E).

### Mutant K63UbR exhibits attenuated response to EGFR, IGF-1R and allosteric AKT inhibition

**Figure 5:**
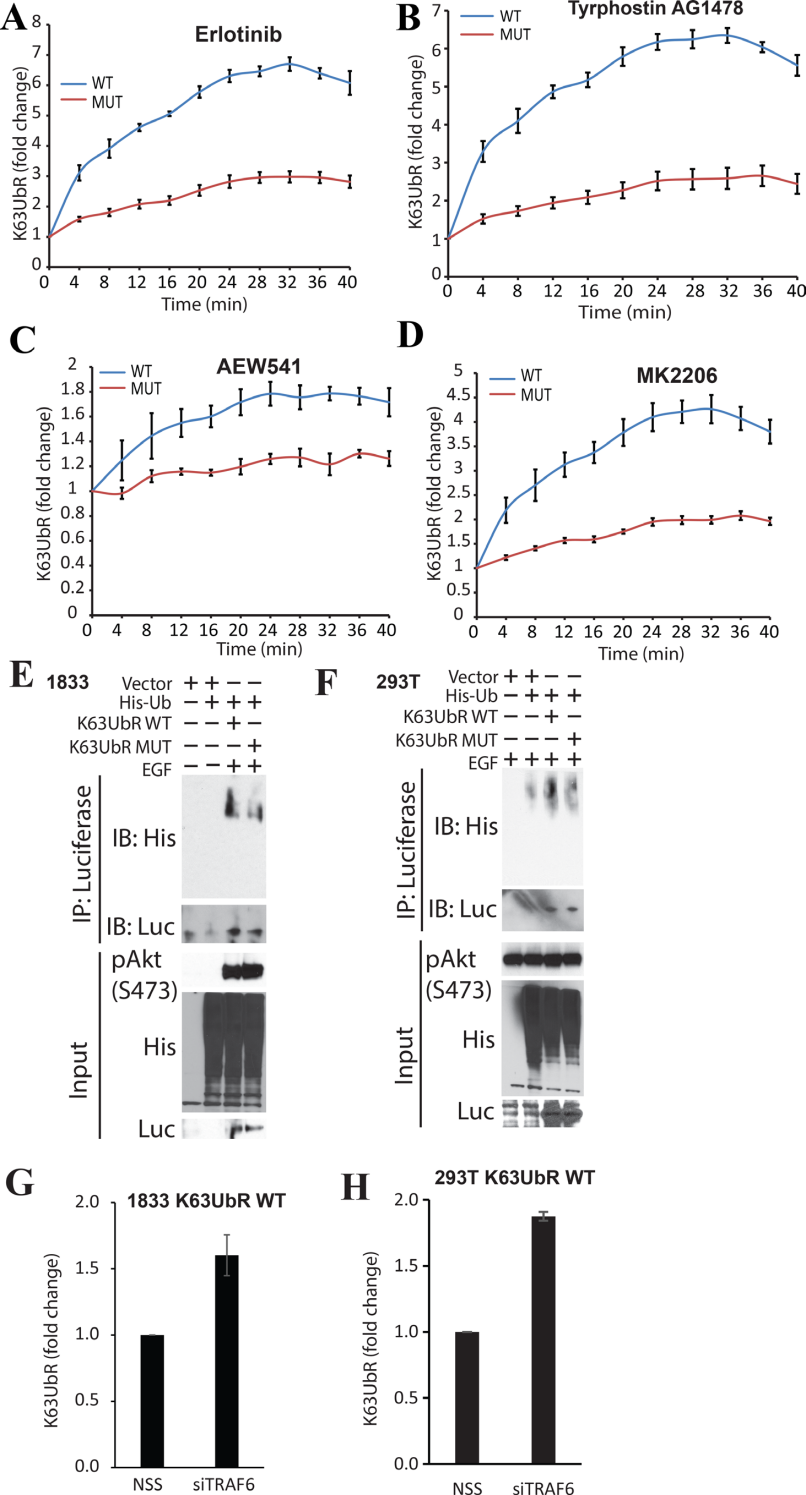
Validation of the specificity of the K63UbR reporter using mutagenesis (**A–D**) To confirm the specificity of the reporter, K63UbR WT and MUT expressing cells were treated with Erlotinib, Tyrphostin AG1478, AEW541, or MK2206 (all 5µM) and reporter activity was measured and plotted as fold change from vehicle (DMSO). The activity with vehicle was set as 1 fold for all time points for each reporter and is not shown on the plots. (**E–F**) To further confirm that only the K63UbR WT reporter gets ubiquitinated, not the MUT, we transfected the K63UbR WT or MUT reporter coding plasmids along with His-tagged Ubiquitin into either MDA-231-1833 or 293T cells. The resulting lysates were immunoprecipitated using Luciferase specific antibody and probed with antisera against his-tag. (**G–H**) MDA-231-1833 or 293T K63UbR WT stable cells were plated in 6-well plate and transfected with non-targeting scramble siRNA (NSS) or siRNA specific to TRAF6 (siTRAF6) in triplicates. 72 hours post transfection luciferin was added and reporter activity was measured, error bars denote SEM.

The specificity of the reporter was further confirmed by constructing a mutant reporter (K63UbR-MUT) wherein both lysine residues within in the AKT substrate peptide were substituted with arginine (K8R, K14R). This mutant peptide should not undergo K63-linked poly-ubiquitination [10, 32] (Figure 1A) thus should not respond to EGFR, IGF-1R or AKT inhibitors. To demonstrate that the K63UbR reporter is specific, bioluminescence activity of the WT and the MUT reporters was evaluated in response to inhibitors of EGFR, IGF-1R, AKT (Figure 5A, 5C, 5D) as well as a receptor tyrosine kinase (Figure 5B). As seen in Figure 5A-5D the K63UbR-MUT did not show a significant response to these inhibitors while the K63UbR-WT does confirming that the K63UbR is very robust and specific.

### K63UbR-WT undergoes polyubiquitination

We expected that the AKT peptide (encompassing K8 and K14) present within the chimeric reporter would serve as a surrogate for the E3-ubiquitin ligase activities for NEDD4, TRAF6 and SCF^SKP2^ and thus should exhibit polyubiquitination by biochemical methods. MDA-231-1833 (Figure 5E) or HEK293T (Figure 5F) cells were transfected with K63UbR WT or MUT expression plasmids along with his-tagged ubiquitin plasmids and treated with EGF. The resulting samples were immunoprecipitated using luciferase-specific antibody and probed using a His-tag specific (for tagged-ubiquitin) antibody which demonstrated that K63UbR WT was poly-ubiquitinated in the presence of EGF stimulation. In contrast, a diminished level of poly-ubiquitination was observed for the mutant K63UbR suggesting that the polypeptide present within the K63UbR serves as a surrogate for AKT.

### K63UbR responds to TRAF6 knockdown

The K63UbR was designed such that it should not only measure the upstream signaling changes (inhibition or activation) but also removal of E3-ubiquitin ligases. To establish that K63UbR measures siRNA mediated knockdown of AKT-specific E3-ubiquitin ligase, MDA-231-1833 K63UbR WT cells were transfected with control (NSS) or TRAF6 siRNA followed by bioluminescence imaging (Figure 5G). An increase in bioluminescence was observed in cells depleted of TRAF6. In agreement, HEK293T-K63UbR cells transfected with NSS (control) or TRAF6 siRNA also exhibited a 2 fold increase in the reporter activity when TRAF6 was knocked down (Figure 5H).

### Polyubiquitination of K63UbR-WT reporter is K63-linkage specific

**Figure 6:**
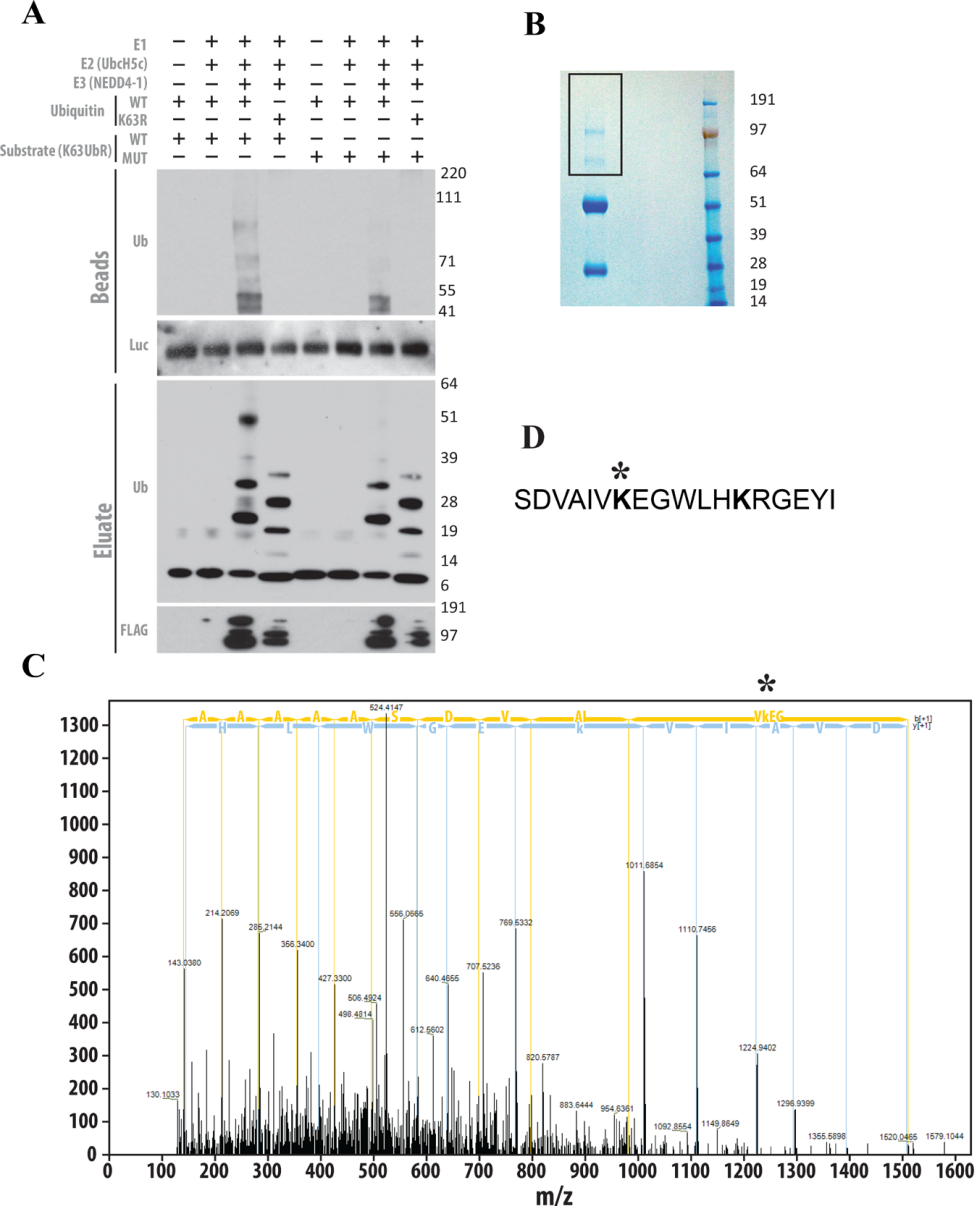
The AKT substrate peptide present within the chimeric K63UbR WT reporter is a suitable target for K63-linkage specific ubiquitination (**A**) The K63UbR WT and MUT reporters were overexpressed in HEK293T cells and immunoprecipitated using luciferase specific antibody. Antibody-protein complex were captured using protein-A/G sepharose beads. The resulting beads were used as substrate in the *in vitro* ubiquitination reactions utilizing bacterially or insect cell purified E1, E2 (UbcH5c) and E3 (NEDD4-1) enzymes in the presence of either WT or K63R mutant ubiquitin. K63UbR WT underwent ubiquitination which was K63-linked (lane 3) as K63R mutant ubiquitin failed to show such higher molecular weight species. In contrast, the K63UbR MUT substrate showed no ubiquitin modifications (lane 7). (**B**) Affinity purified chimeric K63UbR WT reporter was *in vitro* ubiquitinated (similar to lane 3 in Figure 6A) and resolved in SDS-PAGE and cut for processing for MS/MS. (**C**) *In vitro* ubiquitinated K63UbR WT chimeric protein was run on gel and gel slices were cut and digested with trypsin, the peptides were introduced into a high-resolution mass spectrometer (Orbitrap Fusion Tribrid) and MS/MS data were acquired. The MS/MS spectrum indicates that the lysine (K8) in the target sequence (AAAAAAASDVAIVK*EGWLHK; ^*^ ubiquitinated lysine; precursor m/z [M+H]^+4^ = 524.03; Dm = 3.96 ppm) is poly-ubiquitinated by K63-linked chains. Observed *b-* and *y-*ions are indicated. This data confirms that the Lys8 in AKT peptide within K63UbR WT reporter is poly-ubiquitinated by K63-linked chains. (**D**) the location of the ubiquitinated lysine (K8) within the AKT peptide (amino acid 2–19) utilized to create the K63UbR reporter.

We next conducted an *in vitro* ubiquitination followed by Tandem Mass Spectrometry (MS/MS) to investigate if the AKT substrate peptide present within the K63UbR WT reporter undergoes K63-linkage specific poly-ubiquitination. HEK293T cells were transfected with either WT or MUT K63UbR plasmids. Following 24 hours of transfection cell lysates were immunoprecipitated using a luciferase specific antibody. The resulting precipitates were used as substrate in an *in vitro* ubiquitination reaction utilizing bacterial or insect cell purified E1, E2 (UbcH5), E3 (NEDD4-1) and either WT or K63R mutant ubiquitin proteins. The resulting samples were resolved by SDS-PAGE followed by immunoblotting (Figure 6A) to demonstrate that the AKT substrate peptide present within the K63UbR WT and not MUT reporter undergoes poly-ubiquitination and that this ubiquitination is K63 specific as it was not detected when the K63R mutant ubiquitin was utilized in the reaction. In addition, poly-ubiquitination was not detected when the K63UbR MUT reporter was used as substrate in the *in vitro* assay (Figure 6A). Furthermore, to confirm that the AKT target residues present in the K63UbR WT reporter were poly-ubiquitinated at the appropriate residue, *in vitro* ubiquitination reaction were performed as above, resolved on SDS-PAGE and the bands representing the reporter and higher molecular weight poly-ubiquitinated species were excised (Figure 6B) for Tandem Mass Spectrometry (MS/MS) analysis. These analysis, confirmed that the K8 within the target AKT peptide of K63UbR WT underwent ubiquitin-linkage (Figure 6C, 6D).

### K63UbR is suitable for high throughput screens

**Figure 7:**
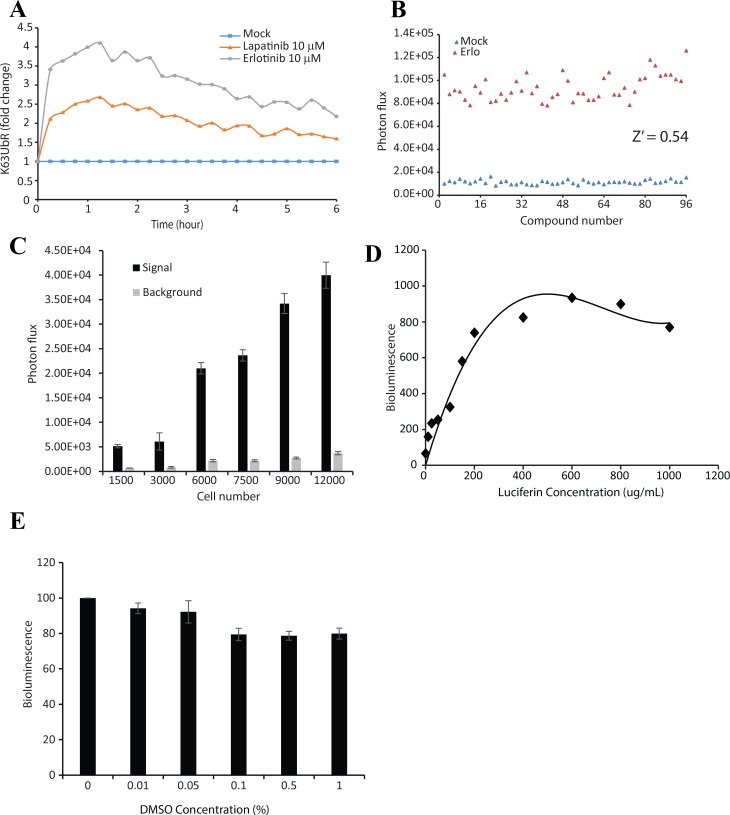
K63UbR is very robust and is suitable for high-throughput screening assays (**A**) MDA-231-1833 cells stably expressing the K63UbR WT were plated in 96-well plates and treated with Erlotinib, Lapatinib or DMSO vehicle and glosensor substrate was added and bioluminescence was acquired every 15 minutes for 6 hours. The data show that the reporter response is very rapid and the reporter activity remains elevated for prolonged period of time. (**B**) MDA-231-1833 cells stably expressing the K63UbR WT were plated in 96-well plates and treated with either vehicle or 5 µM Erlotinib in a checker-box pattern. Bioluminescence was acquired 30 minutes after treatment. 5 µM Erlotinib significantly activated the reporter (Z’ = 0.54) confirming the suitability of the reporter for HTS assays. (**C**) The effect of seeding density on signal and background was estimated in 96-well plates. Cells were seeded at 1500, 3000, 6000, 7500, 9,000, and 12,000 cells/well. Signal and background represent MDA-231-1833 K63UbR WT cells incubated with Erlotinib (10 µM, signal) or DMSO (vehicle, background). Values represent the mean ± SEM of 2 separate experiments, each with 8 replicates. (**D**) Effect of luciferin concentration on cellular response was estimated by plating K63UbR WT cells and treating with multiple doses of luciferin. The cells were imaged 5 to 10 min following substrate addition. (**E**) The effect of DMSO concentration on signal generation was estimated by plating 6000 cells/well 48 hours prior to assay. On the day of assay, cell were treating with increasing concentrations of DMSO for 5-10 minutes, luciferin substrate was added and the resulting photon counts were acquired and plotted.

Since K63 poly-ubiquitination of AKT provides a novel and biologically significant target for drug development, we evaluated the suitability of the K63UbR reporter in the context of a high throughput screen. K63UbR expressing cells were treated with EGFR inhibitors (Erlotinib or Lapatinib) and the reporter activity was measured every 15 minutes from the same plate, which confirmed that the reporter is very robust and maintains activity for several hours (Figure 7A). Next, reporter expressing cells were treated with EGF in the presence of Erlotinib or DMSO in 96-well plates. A Z’ of 0.54 was achieved (Figure 7B) confirming the suitability of the reporter for high-throughput screening campaigns. Furthermore, the effect of cell density on signal to background ratio (S/B) was measured by plating cells at different densities (1500, 3000, 6000, 7500, 9000 and 12000) in 96-well plates and treating with vehicle (DMSO, background) or Erlotinib (10 µM, signal) (Figure 7C). A constant signal at all cell densities tested was observed, suggesting that K63UbR S/B is minimally affected by cell density. Additionally, the effect of luciferin concentration on reporter signal was accessed to define optimal assay conditions (Figure 7D). We identified that 100 *µ*g/mL luciferin yielded a stable bioluminescence signal. Since the most commonly used solvent for small molecule libraries is DMSO, we confirmed that the DMSO concentrations (up to 1%) had little effect on K63UbR reporter activity (Figure 7E). The data presented demonstrate that the reporter maintained above 85% basal activity up to 1% DMSO concentration.

The findings described above provide evidence that the K63UbR is a specific, and sensitive reporter for quantitative, dynamic and non-invasive monitoring of AKT specific K63 poly-ubiquitination in live cells and is suitable for live cell high-throughput screens.

## DISCUSSION

Although a critical role of K63-linked ubiquitination and deubiquitination in the activity of oncogenic kinases is appreciated in tumorigenesis [10–14], technologies to specifically monitor this key event under physiological conditions have been missing. Most assays that monitor E3-ubiquitin ligase activities [44, 45] rely on the use of purified proteins, therefore factors such as the selection of an active ubiquitin-conjugating enzyme (E2), the need of other accessory proteins for formation of an active complex, and optimization of the active complex components needs to be empirically determined for optimal functioning in a purified system.

Most studies to measure ubiquitination in live cells by fluorescence [46] or bioluminescence [47, 48] have focused on the ubiquitin-proteosome system (UPS). Reporters for imaging non-proteosomal ubiquitination events have been described; however, they either work in a cell free system where pure components need to be supplied [49, 50] or they are based on fusing the ubiquitin binding domain (UBD) to fluorescent proteins [39, 51, 52]. Therefore, they monitor total pools of the K63-ubiquitin chains in cells, and not target-specific E3-ubiquitin ligase activity. In addition, due to reliance on fluorescence, these reporters cannot be easily adapted for quantitative studies in deep tissues (live mouse models) and may not be amenable to high-throughput systems. A recent study describing a reporter for E3-ubiquitin ligase specific activity in live cells is based on the MDM2-luciferease fusion protein which measures MDM2 auto-ubiquitination leading to its degradation [53]. This reporter may have several limitations: (i) since it monitors proteosomal degradation, it is an indirect measure of E3-ubiquitin ligase activity in live cells, (ii) it is specific for an E3-ubiquitin ligase and not for the substrate which may be ubiquitinated by additional E3-ligases as in the case of AKT.

The K63UbR overcomes these challenges by utilizing a K63-specific substrate sequence (e.g. the AKT PH-domain) as well as a K63-specific UBD (Ubiquitin Binding Domain) [37, 52], flanked by split luciferase within the chimeric reporter molecule. This technologically advanced molecular imaging reporter (K63UbR) allows direct, quantitative, dynamic, and sensitive measurement of substrate specific E3-ubiquitin ligase activity in live cells. Additionally, it is an activatable reporter where inhibition of E3-ubiquitin ligase activity or activation of a deubiquitinase leads to an increase in reporter activity, thus non-specific cytotoxic conditions would not yield false positives. Moreover, due to the activity dependent reconstitution of luciferase fragments, rather than accumulation, this reporter system may yield faster kinetics (Figures 2B, 2F, 2J, 3A, and 3E).

Although the role of Lys63-linked poly-ubiquitination in AKT activation has been established, the exact mechanism by which growth factor receptors such as EGFR and IGF-1R, signal activation of E3-ubiquitin ligases (TRAF6, SKP2/SCF, or NEDD4-1) activity towards AKT, is still unknown. Additionally, there are other signaling pathways such as integrin, cytokine receptor, GPCR, CDK2/cyclin A, and CD-40 [54–58] that activate AKT and most likely also require K63–linked polyubiquitination of AKT, the mechanism of which is not understood. Therefore, methods for measuring changes in ubiquitination status in a target specific manner, in live cells, would lead to an improved understanding of these signaling pathways. One of the limitations of our study is that we established reporter expressing stable cell lines and validated in a single breast cancer line (MDA-231 derived 1833) and a non-cancer cell line (HEK293T; see Figure 5F and 5H). Based on our prior experience with other split-luciferase based reporters [59–62], we believe that this reporter will be amenable for use in additional cell lines. In addition, although we have not evaluated the reporter *in vivo* using tumor xenograft mouse models, the strength of luciferase based reporters is that they are easily adapted for *in vivo* studies due to the depth of signal penetration of bioluminescence.

One needs to establish stable cell lines and screen multiple single-cell clones to identify clones which express reporter at an optimal level to yield the best sensitivity, dynamic range and signal/background ratio as this reporter requires intra-molecular complementation of the luciferase fragments in response to signaling cues, and cells that express high levels of the reporter result in a high background due to inter-molecular complementation. Our prior work demonstrating the adaptability of luciferase complementation assays to monitor proteolytic activities and kinase activity (tyrosine and serine/threonine) [59–62], suggests that K63UbR will serve as a prototype and can be easily adapted for the development of additional reporters for other E3-ubiquitin ligase activities.

## MATERIALS AND METHODS

### Selection of the substrate, Ubiquitin binding domain and construction of the reporter

This reporter consists of a K63-linkage specific polyubiquitination target sequence of AKT (amino acid 2-19 of the PH domain harboring Lys8 and Lys14) [10, 31, 32]. Based on the fact that the selected short peptide of AKT is very specific and is present only in AKT1 (Entrez BLAST search), short peptides can be ubiquitinated *in vitro* [51], and have surrogated for endogenous proteins in kinase reporters [60, 62], we chose this sequence for construction of the reporter. Several K63-linkage specific tandem ubiquitin interaction motif (tUIMs) with varying degrees of selectivity have been identified [33–37]. The tUIM with multiple NZF are known to possess remarkable preference for binding to K63-linked polyubiquitin chains [37]. We used ubiquitin interaction motifs (tUIM) from RAN-binding domain containing 1 (ZRANB1/Trabid; aa 2-200) containing three tandem Npl4 related zinc finger (NZF) motifs for construction of the reporter. These sequences are flanked by the N-terminus of luciferase (N-Luc, 4-354) at the carboxyl-terminus of the reporter while the C-terminus of luciferase (C-Luc, aa 358-544) is at the amino-terminus of the reporter. A c-DNA plasmid for ZRANB1 was purchased from Origin Technologies (pEZ-M12-ZRANB1, EX-H4597-M12). A seven amino acid long poly Alanine linker shown to increase the specificity of the synthetic UBD to K63-linkage specific chains [39] was inserted between the UBD and the AKT substrate peptide sequence. The BTR reporter [62] was re-cloned into the plasmid harboring the above mentioned luciferase fragments and was opened with NotI-HF and XmaI and cipped using calf intestinal alkaline phosphatase (New England Biolabs). The PCR fragment containing the linkers, NZF domain, poly A linker, AKT substrate peptide was also digested with NotI-HF and XmaI and ligated using the Quick ligation kit (Roche) and transformed into XL-10 Gold cells (Invitrogen) and spread on agar plates containing ampicillin (50 µg/mL). The following day eight colonies were picked, confirmed by restriction digestions, and by sequencing. The mutant reporter, K63UbR-MUT, was generated by substituting both Lysins within the PH domain with arginine (K8R, K14R) using a single primer mutagenesis protocol, with minor modifications as described earlier. The reporter plasmids will be freely available to researchers upon request to the corresponding authors.

Initial model of the chimeric reporter was built using the I-TASSER server [63]. Manual modeling operations were performed in Coot [64] and molecular graphics were generated in Pymol [65] [Available at: http://www.pymol.org].

### Reagents and reagent preparation

The primers were synthesized and PAGE purified by IDT DNA. Fugene 6 transfection reagent was purchased from Promega while Lipofectamine RNAiMax was from Life Technologies/Invitrogen. All cell culture reagents including media, antibiotics were from GIBCO/Invitrogen. Antibodies to total Ubiquitin (clone P4D1) was from Santacruz Biotechnology, Luciferase (Millipore), pAKT, AKT (Cell Signaling), Ubiquitin, Lys63 specific (clone Apu3, Millipore), His-tag (clone H3 and C-term, Invitrogen) or Millipore (H8 clone). FLAG tag-HRP antibody (Clone M2) was purchased from Sigma Aldrich. HRP-conjugated and fluorophore-conjugated secondary antibodies were from Jackson ImmunoResearch. ON-TARGET plus siRNA to TRAF6 and scrambled control siRNA were obtained from GE Life Sciences/Dharmacon. D-Luciferin was from Xenogen Corp while GloSensor was from Promega. Erlotinib was a kind gift from Genentech. Tyrphostin AG1478 was obtained from Cayman Chemical. TNF-α antagonist (WP9QY) and IL-1R antagonist were from SantaCruz biotechnology. Recombinant IGF-1, IL-1α were from PepreoTech. CI-1040, GF109203X, NVP-AEW541 and MK2206 (Cayman Chemical), and Linsitinib (LC Laboratories). Protein A and Protein G sepharose beads were from GE HealthCare. Purified NEDD4-1 (Sigma Aldrich), Myc-tagged ubiquitin (Cat. No. U-115) UBE1 (Cat. No. E305), and UBCH5 (Cat. No. E2-616) all were from Boston Biochemicals.

### Cell Culture, transfection and generation of stable cell line

HEK293T cell line was obtained from ATCC while a metastatic breast cancer cell line 1833 [66] derived from MDA-MB-231 was provided by Dr. Joan Massague. Both the cell lines were maintained in DMEM supplemented with 10% heat-inactivated fetal bovine serum, 1% glutamine, and 0.1% penicillin/streptomycin/gentamycin. Cell cultures were grown in a humidified incubator at 37° C and 5% CO_2_. Stable cell lines were developed by transfecting the K63UbR reporter plasmids into 1833 and HEK293T cells using Fugene 6 and resulting clones were selected using 250 µg/mL (1833) or 500 µg/mL (HEK293T) G418 containing media for 15-20 days. Twelve stable clones were picked for both wild-type and mutant cell lines. The K63UbR-WT and MUT expressing clones were analyzed by bioluminescent imaging following treatment with 5 µM Erlotinib. Three stable cell lines for each reporter were expanded, frozen and maintained in 250 µg/mL G418 containing media.

### Treatments, bioluminescent assays and live-cell imaging

For the reporter assay, 1833 and 293T K63UbR cell lines were seeded in 96-well (5 × 10^3^ cells/well), black-walled, clear bottom plates (Corning, Inc.) 24-48 hours prior to assaying. Cells were treated in serum-free media with varied concentrations of test compounds (Erlotinib, NVP-AEW541, Linsitinib, MK2206, Tyrphostin AG1478, TNF-α antagonist, IL-1R antagonist) in presence of respective growth factors (TNFα, EGF or IGF-1). D-luciferin or GloSensor (100 µg/ml final concentration) was added to cells right away, and photon counts for each condition were acquired 1 minute after incubation with the substrate using an IVIS 200 imaging system (Xenogen). Alternatively cells were plated in white walled, clear bottom plates and luminescence was read with an Envision 2104 multi-label plate reader (Perkin Elmer) after addition of GloSensor (100 µg/ml final concentration) to the cell medium; each experiment was done at least in quadruplicate and repeated at least three times. For the high throughput screen, 10 µl of an intermediate DMSO stock of each compound in the kinase inhibitor library was added to the cell medium using a Beckman Biomek NX^P^ Laboratory Automation Workstation (Beckman Coulter Inc.), yielding a final assay concentration of 5 µM for each compound; all appropriate controls were included and cells were incubated for 5-10 minutes before GloSensor substrate was added and bioluminescence measured.

### siRNA transfection

Knockdown experiments were performed by transfecting 1833 and 293T K63UbR WT reporter cells with 100 nM small interfering RNA (siRNA) for TRAF6 and non-silencing siRNA (NSS) as a negative control using Lipofectamine RNAiMax. The transfected cells were incubated for 48-72 hours, serum starved for 3-4 hours, and 50 ng/mL IGF-1 or 100 ng/mL EGF added just prior to evaluating reporter activity with bioluminescent imaging and Western analysis. Fold change in reporter activity was calculated over change in activity in NSS transfected cells.

### Protein expression in 293T cells and immunoprecipitation

HEK293T cells were transfected with 2 µg plasmid (K63UbR WT and MUT) using Fugene 6. Forty eight hours after transfection, cells were washed in cold PBS and lysate was made in IP lysis buffer (50 mM Tris PH 7.4, 150 mM NaCl, 0.25% Deoxycholate sodium salt, 1% NP40, 5% Glycerol, and 1 mM EDTA) supplemented with 1X PhosStop (Roche), Sodium Ortho Vanadate, Sodium fluoride, PMSF, and β-Glycerol phosphate. Immunoprecipitation of the K63UbR reporter molecule was carried out by incubating cell lysate (2500 µg protein) with 15 µg luciferase specific antibody over night at 4 C. The immune complex was captured using 20 µl slurry of protein A/G-coupled Sepharose beads for 1 hour, washed three times with IP-lysis buffer and two times with *in-vitro* ubiquitination buffer (250 mM Tris PH 7.5, 50 mM MgCl2, 50 mM DTT, and 20 mM ATP). The washed beads were used as the substrate for subsequent *in vitro* ubiquitination reactions.

### *In vitro* ubiquitination reaction

The *in vitro* ubiquitination reaction was carried out in a 15 μl reaction volume in *in-vitro* ubiquitination buffer, 10 μg of Myc-tagged ubiquitin, 0.35 μg of UBE1, and 0.5 μg of UBCH5 and 0.1 μg NEDD4-1. The reaction mixtures were incubated at 37° C for 2 hours. The reaction was terminated after boiling with 4× Nupage sample buffer and were boiled for 10 min at 100° C. Reactions were resolved on SDS-PAGE gels and transferred to PVDF membranes. Membranes were probed against specific primary antibodies (Ubiquitin, luciferase or Flag-tag for NEDD4-1) followed by HRP conjugated secondary antibodies then visualized using the Enhanced Chemiluminescence (ECL) Western Blotting System (GE Healthcare).

### MASS-SPEC analysis for validation of the K63UbR WT

The *in vitro* ubiquitination sample of the WT reporter (K63UbR WT) was run on 4-12% Bis-Tis gel in clean conditions and stained using the MASS Spec compatible SimplyBlue SafeStain (Invitrogen). The protein samples were processed at the Proteomics Resource Facility of the Department of Pathology at the University of Michigan. Gel slice corresponding to (K63UbR) was destained with 30% methanol for 4 h. Upon reduction (10 mM DTT) and alklylation (65 mM 2-Chloroacetamide) of the cysteines, proteins were digested overnight with sequencing grade, modified trypsin (Promega). Resulting peptides were resolved on a nano-capillary reverse phase column (Acclaim PepMap C18, 2 micron, 25 cm, ThermoScientific) using a 1% acetic acid/acetonitrile gradient at 300 nl/min and directly introduced in to Orbitrap Fusion tribrid mass spectrometer (Thermo Scientific, San Jose, CA). MS1 scans were acquired at 120 K resolution. Data-dependent high-energy C-trap dissociation MS/MS spectra were acquired with top speed option (3 sec) following each MS1 scan (relative CE ∼32%). Proteins were identified by searching the MS/MS spectra against human protein database (UniProtKB; 4-16-2015) appended with chimeric K63UbR sequence using Proteome Discoverer (v2.1, Thermo Scientific). Search parameters included MS1 mass tolerance of 20 ppm and fragment tolerance of 0.2 Da; two missed cleavages were allowed; carbamidimethylation of cysteine was considered fixed modification and oxidation of methionine, di-glycine remnant on lysine were considered as potential modifications. Percolator algorithm was used for discriminating between correct and incorrect identifications. Proteins/peptides that were identified with <1% false discovery rate (FDR) were retained.

### Data analyses

Pearson correlation coefficient (r) was estimated to confirm how well the Erlotinib, Linsitinib and AEW541 doses correlate to K63UbR reporter fold induction. Analysis of statistical significance (student’s *t*-test, *p* values) was performed to estimate the significance of reporter fold induction with various doses. Additionally, regression analysis (goodness of fit; R^2^) was carried out to confirm relationships between doses, and reporter fold induction. The EC50 values for different drugs were estimated on GraphPad Prism.

## Abbreviations

UBD: ubiquitin binding domain
HTS: high throughput screen
UIM: ubiquitin interaction motifs
DUB: deubiquitinase
EGF: epidermal growth factor
IGF1: insulin like growth factor 1
TNF-α: Tumor Necrosis Factor-alpha
IL1-R: Interleukin 1-receptor
TRAF6: TNF receptor associated factor 6
SKP2: S-phase kinase associated protein 2
NEDD4: neural precursor cell expressed, developmentally down-regulated 4, E3 ubiquitin protein ligase
DMSO: dimethyl sulfoxide
